# Evaluating Anti-CD32b F(ab) Conformation Using Molecular Dynamics and Small-Angle X-Ray Scattering

**DOI:** 10.1016/j.bpj.2018.03.040

**Published:** 2018-07-17

**Authors:** Emma J. Sutton, Richard T. Bradshaw, Christian M. Orr, Bjorn Frendéus, Gunilla Larsson, Ingrid Teige, Mark S. Cragg, Ivo Tews, Jonathan W. Essex

**Affiliations:** 1 Antibody & Vaccine Group, Cancer Sciences Unit, Centre for Cancer Immunology, Faculty of Medicine, University of Southampton, Southampton General Hospital, Southampton, United Kingdom; 2 Department of Chemistry, University of Southampton, Highfield Campus, Southampton, United Kingdom; 3 BioInvent International AB, Lund, Sweden; 4 Department of Biological Sciences, Institute for Life Sciences, University of Southampton, Highfield Campus, Southampton, United Kingdom

## Abstract

Complementary strategies of small-angle x-ray scattering (SAXS) and crystallographic analysis are often used to determine atomistic three-dimensional models of macromolecules and their variability in solution. This combination of techniques is particularly valuable when applied to macromolecular complexes to detect changes within the individual binding partners. Here, we determine the x-ray crystallographic structure of a F(ab) fragment in complex with CD32b, the only inhibitory Fc-*γ* receptor in humans, and compare the structure of the F(ab) from the crystal complex to SAXS data for the F(ab) alone in solution. We investigate changes in F(ab) structure by predicting theoretical scattering profiles for atomistic structures extracted from molecular dynamics (MD) simulations of the F(ab) and assessing the agreement of these structures to our experimental SAXS data. Through principal component analysis, we are able to extract principal motions observed during the MD trajectory and evaluate the influence of these motions on the agreement of structures to the F(ab) SAXS data. Changes in the F(ab) elbow angle were found to be important to reach agreement with the experimental data; however, further discrepancies were apparent between our F(ab) structure from the crystal complex and SAXS data. By analyzing multiple MD structures observed in similar regions of the principal component analysis, we were able to pinpoint these discrepancies to a specific loop region in the F(ab) heavy chain. This method, therefore, not only allows determination of global changes but also allows identification of localized motions important for determining the agreement between atomistic structures and SAXS data. In this particular case, the findings allowed us to discount the hypothesis that structural changes were induced upon complex formation, a significant find informing the drug development process. The methodology described here is generally applicable to deconvolute global and local changes of macromolecular structures and is well suited to other systems.

## Introduction

Monoclonal antibodies (mAb) with their high specificity and ability to engage immune effector mechanisms are revolutionizing the treatment of diseases such as cancer and autoimmune conditions (1, 2). Currently, the majority of clinically approved mAb are of the immunoglobulin G (IgG) class (3). The structure of IgG is critical for its function; variable regions within the F(ab) domains confer specificity, whereas the Fc domain allows interaction with Fc*γ* receptors (Fc*γ*R) on the surface of immune cells to elicit effector functions (4). Recent work has focused on Fc engineering to augment cellular interactions and therapeutic responses (5); however, it is evident that epitope specificity and antibody conformation also have major implications for biological outcomes (6, 7, 8, 9).

Small-angle x-ray scattering (SAXS) has become a popular method to study flexible macromolecules and can provide crucial insight into the conformation and behavior of proteins in solution (10, 11). As the spatial resolution of SAXS is limited, many programs exist to allow comparisons of crystallographic structures to SAXS data (12, 13, 14). However, often the crystal structure of a protein does not accurately represent the conformation(s) seen in solution (15, 16), so a combination of x-ray crystallography and complementary solution-based data are needed to better describe biomolecular structure. Reliable methods able to bridge the gap between high-resolution atomic structures and physiologically relevant SAXS data are therefore required to fully understand the structure-function relationships driving mAb activity.

In previous work, we identified agonistic and antagonistic antibodies specific to Fc*γ*RIIb (CD32b), the sole inhibitory Fc*γ*R in humans (17). These antibodies are capable of either activating or blocking the receptor, respectively. Dysregulation of CD32b is implicated in cancer and autoimmune conditions (18, 19, 20), making it an attractive target for immunotherapy. How these anti-CD32b antibodies evoke their opposing effects is currently unclear and requires knowledge of mAb structure and conformation, both alone and in complex with CD32b, in addition to characterization of binding epitopes and interactions.

Here, we compare the F(ab) domain of the anti-CD32b mAb, 6G08, extracted from a CD32b:6G08 crystal complex, with SAXS data for the F(ab) alone in solution to investigate potential conformational changes between the free and bound forms. The theoretical scattering profile for the F(ab) extracted from the crystal complex, generated using CRYSOL (12), shows poor agreement to the solution-phase SAXS data as assessed by the *χ*^2^ fit between the two curves, suggesting a conformational change in the F(ab) upon binding. High-resolution methods are therefore required to identify the structural differences between the 6G08 F(ab) crystal structure and the 6G08 F(ab) SAXS data.

Using long-timescale molecular dynamics (MD) simulations in explicit solvent, we investigate the dynamics of the 6G08 F(ab) in solution at atomistic resolution. Scattering curves for frames extracted throughout MD simulations were generated in CRYSOL and used to assess agreement of the MD structure to the 6G08 F(ab) SAXS data. Through dimensionality reduction via principal component analysis (PCA) combined with *χ*^2^ scoring, we are able to identify specific structural characteristics that directly influence the extent to which atomistic MD structures agree with the experimental data for the F(ab). This complementary use of simulation and experimental approaches provides a method that will help identify key structural behaviors that govern the agonistic or antagonistic characteristics of anti-CD32b antibodies.

## Materials and Methods

### Sample preparation

The antigen-binding F(ab) fragment (residues 1–439) of the anti-CD32b antibody, 6G08, and the extracellular domain of CD32b (residues 43–217) were produced as previously described (17) and supplied by BioInvent International (Lund, Sweden). For SAXS experiments, 6G08 F(ab) was purified via size exclusion gel filtration using a Superdex 200 10/300 GL column. Samples were eluted in buffer containing 50 mM HEPES, 150 mM KCl at pH 7.5. Flow through buffer was retained and stored with protein samples for data collection.

### Crystallographic structure determination

For structure determination, the complex between CD32b and 6G08 F(ab) was formed by incubation at room temperature (30 min) followed by size exclusion chromatography using an S200 10/300 GE column (General Electric Healthcare, Little Chalfont, United Kingdom). The final complex was analyzed by high-performance liquid chromatography, concentrated to 7.1 mg/mL and crystallized using the PACT premier screen (21) and an Art Robbins Gryphon (Art Robbins Instruments, Sunnyvale, CA). Crystals appeared in condition B12, containing 20% polyethylene glycol 6000 buffered with 0.1 M MES and 0.01 M zinc chloride at pH 6.0, within a week and were used for diffraction experiments at the European Synchrotron Radiation Facility ID23-2 at 70 K using a wavelength of 0.9763 Å, giving the final data set in space group P3121 to a resolution of 2.99 Å. All data manipulation was carried out with software from the CCP4 suite (22). Molecular replacement with Molrep (23) used Protein Data Bank (PDB): 3H42 as a search model for the F(ab) fragment (24) and PDB: 1H9V for CD32b (25). The F(ab) fragment was rebuilt as the amino acid sequence was corrected from the initial molecular replacement model. Rebuilding of all chains was conducted in coot (26), and refinement in Refmac5 (27) was cycled. The final model included glycosylation for CD32b with one N-acetyl-glucosamine residue linked to each Asn106 and Asn187. The final model contained 4063 atoms, one phosphate, and 21 water molecules and was refined to an R-factor of 19.41 with an R free of 23.19. The Ramachandran analysis indicated the side chains were 90.17% in favored regions. Further refinement statistics are available in Table S1. The final structure was deposited in PDB: 5OCC.

### SAXS data collection and primary analysis

The purified 6G08 F(ab) was concentrated to 5 mg/mL using a 10,000 molecular weight cutoff polyethersulfone membrane Vivaspin 2 centrifugal concentration device. After concentration, samples were diluted 1:2 using flow through buffer to create a concentration series. Data sets were collected at the European Synchrotron Radiation Facility on BM29. Scattering was defined as a function of the momentum transfer, *q*:

(1) $$q=\frac{4\pi \;\sin\;\theta }{\lambda },$$

where 2*θ* is the scattering angle and *λ* is the wavelength of the incidence beam (0.99 Å). Samples were loaded using the automated sample changer (28), and data were acquired at 20°C. For each sample, 10 frames with a 2 s exposure time were collected and automatically assessed for radiation damage, and then an average profile generated. Scattering from buffer samples was subtracted from the corresponding protein sample to generate the SAXS scattering profiles.

Primary data analysis was conducted in Primus (29) and ScÅtter (version 3, R. Rambo), during which the radius of gyration (R_g_) and maximal dimension (D_max_) values were calculated from the SAXS data. The scattering curves in addition to the R_g_ and D_max_ values for each of the 6G08 samples were compared to ensure consistency between concentrations, and then a merged curve across the sample concentrations was generated and used for all further data analysis.

### Comparison of atomistic structures to SAXS data

To compare the agreement between atomistic structures and SAXS data for the 6G08 F(ab), scattering profiles were generated using CRYSOL version 2.8.3 and compared to the experimental SAXS data. The initial comparison of the 6G08 F(ab) crystal structure to the SAXS data was performed with CRYSOL using the constant subtraction fitting parameter to take into account potential errors associated with buffer subtraction in the experimental data (12). All subsequent fitting calculations were then conducted in CRYSOL using a truncated SAXS data set with a maximal *q* value of 0.2 Å^−1^. Agreement between the scattering curve for the atomic structure and the experimental scattering data was assessed via *χ*^2^ score. For the purpose of this study, a structure with a *χ*^2^ score <3 was considered to have good agreement with the 6G08 F(ab) SAXS data. Full details on the scattering calculation and fitting procedures are available in the Supporting Materials and Methods, Text S2 and Table S2. All figures showing SAXS data were made in gnuplot with scattering intensity plotted as log I(q) to the base 10 vs. q (Å^−1^). Residuals for the SAXS fits were defined as log I(q)_exp_ – log I(q)_mod_, where I(q)_exp_ refers to the experimental scattering intensities and I(q)_mod_ refers to the theoretical scattering intensities calculated for the atomic structure. Elbow angles for atomistic structures were calculated using PyMOL (30).

### MD simulations

MD simulations were performed using Amber 16 software to generate atomistic configurations of the 6G08 F(ab) fragment in solution (31). For each simulation, the starting structure of the F(ab) fragment was taken from the crystal structure of the 6G08 F(ab) in complex with CD32b. Modeler version 9.17 was used to build in an additional serine residue that was not resolved in the crystal structure of the complex at the C-terminal end of the F(ab) light chain constant domain (32). Positions of the remaining atoms were not altered during the rebuilding process. The F(ab) structure was protonated at pH 7.0 using the online H++ server, version 3.2 (33), resulting in glutamate, aspartate, lysine, and arginine side chains in their standard ionized states and all histidines singly protonated at the epsilon nitrogen. The protonated structure was then solvated in a box of pre-equilibrated TIP3Pwater molecules (34) with each box side made ∼112 Å. After neutralization with chloride ions, additional Na^+^ and Cl^−^ ions were added to achieve a final concentration of 150 mM NaCl (37,895 water molecules with 106 each of Na^+^ and Cl^−^ ions).

Protein and ions were represented with the Amber ff14SB force field (35) and the parameters of Joung and Cheatham (36), respectively. The system was equilibrated to 300 K and 1 bar followed by 1 *μ*s of simulation. Three independent repeat simulations were performed using a different random seed for the Langevin thermostat and randomized initial NaCl ion positions. Structures were extracted from the simulations at 1 ns intervals, giving a total of 3000 structural snapshots for analysis. Full details of simulation protocol are available as Text S1.

### PCA

PCA was performed using the 3000 structures extracted from the three separate simulations. PCA was conducted using the Bio3D R package (37, 38). Translation and rotation of the molecule between frames was removed through an alignment of the protein C*α* atoms in the constant domains of the F(ab). A 3 *N* dimensional covariance matrix was then constructed from the coordinate variations of the C*α* atoms across all frames of the MD trajectory. Diagonalization of this matrix led to 3 *N* eigenvectors and associated eigenvalues defining the principal components of the overall variance in the C*α* position. To allow comparisons between structures from different regions of the PCA space, the MD trajectories were sorted into representative clusters using cpptraj, which is part of the Amber software package. Frames were sorted using the hierarchical agglomerative clustering algorithm with an epsilon distance metric of 2 Å according to a root mean-square displacement alignment on the constant domains of the F(ab). Representative frames were identified for each cluster, allowing comparison of clusters based on the comparison of single MD frames. Full details of cluster size and representative structures, are available in Table S3.

### Intensity difference matrices

In addition to visual comparison, we calculated difference matrices to identify the contributions of specific atoms to the overall scattering intensity, allowing quantitative identification of how regions of differing conformation contribute differently to the SAXS profile. For a particular set of atomic coordinates at a given momentum transfer vector *q*, the total scattering intensity *I*_*q*_ can be calculated as the sum of pairwise interactions via the Debye formula for spherical bodies (39):

(2) $$I_{q}=\sum_{i=1}^{N}\sum_{j=1}^{N}f_{i,q}f_{j,q}\frac{\sin\left(qr_{ij}\right)}{qr_{ij}},$$

where the double sum is performed over all atoms, *r*_*ij*_ is the distance between atoms *i* and *j*, and *f*_*i,q*_ and *f*_*j,q*_ are respectively the atomic form factors for atoms *i* and *j* at the given value of *q*. Explicitly calculating the pairwise double sum over all atoms rather than using a fast approximation to the Debye formula for SAXS profile prediction (14, 40, 41, 42, 43) allows decomposition of the total scattering intensity into contributions from individual atom-atom interactions as a matrix of individual atom-atom intensities. Subtraction of these matrices for two related structures leads to an intensity difference matrix, providing a quantitative view of the contribution of individual differences to the overall scattering profiles.

The calculation of intensity difference matrices was performed using an in-house Python script available at the Zenodo data repository (44). Residue-level form factors with inter-residue distances defined by C*α* positions were calculated using the *ffgen* program (45) and extrapolated to a given *q*-value (14). Difference matrices using heavy-atom or all-atom form factors were seen to highlight identical areas of conformational difference (data not shown); thus, only residue-based form factors were used for computational expediency.

## Results

### Crystalline and solution structure determination

**Figure 1:**
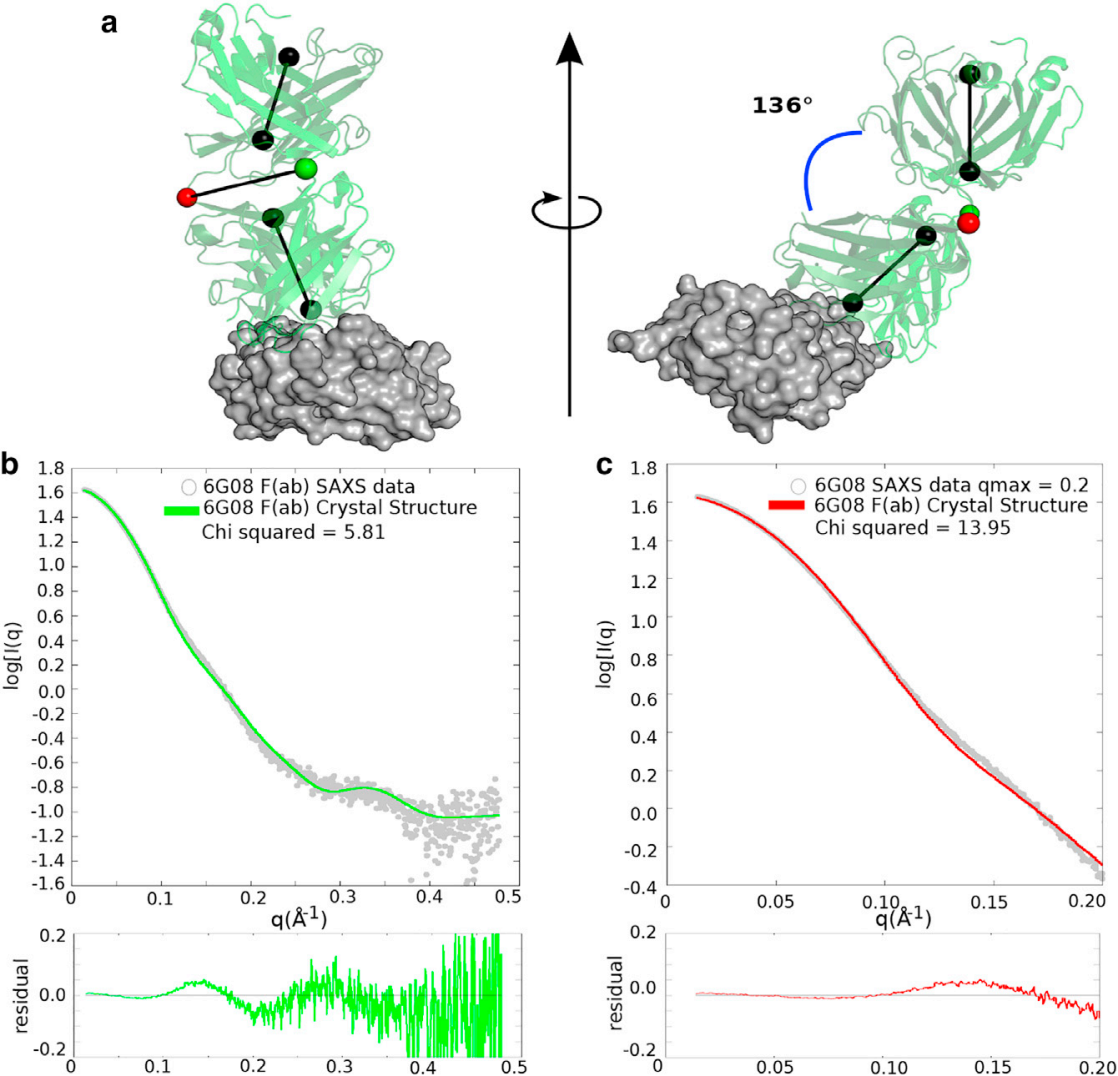
Comparison of the 6G08 F(ab) from the 6G08:CD32b crystal complex to SAXS data for the 6G08 F(ab) alone. (*a*) The crystal structure of the 6G08 F(ab) (*green*) in complex with CD32b (*gray*) is shown. Elbow angle between constant and variable regions is measured between the twofold pseudo symmetry axes of each domain (*black*), with domains split at the residues denoted by the red and green balls for the heavy and light chains, respectively. The two views are rotated around a vertical axis in the paper plane. (*b*) The experimental scattering intensity profile of 6G08 F(ab) (*gray*) is overlaid with the theoretical scattering profile calculated for the 6G08 F(ab) structure from the crystal complex (*green*). (*c*) The 6G08 F(ab) crystal structure is compared to a truncated SAXS data set with a maximal *q* value of 0.2 Å^−1^. The bottom panels (*b* and *c*) show the residual plots for the respective fits; with the residuals defined as log I(q)_exp_ – log I(q)_mod._*χ*^2^ scores are calculated in CRYSOL. To see this figure in color, go online.

Understanding how anti-CD32b mAbs invoke their biological activity requires knowledge of structural changes that occur upon antibody binding to the receptor. To investigate the interaction between the agonist 6G08 F(ab) and CD32b, the structure of the 6G08 F(ab) in complex with the receptor was determined by x-ray crystallography, shown in Fig. 1 *a* (PDB: 5OCC). The crystal structure reveals key interactions between complementarity determining regions (CDRs) 2 and 3 of the F(ab) heavy chain and CDRs 2 and 3 of the light chain with receptor residues previously identified to be involved in binding the Fc domain of IgG (Fig. S1). This supports our previous work demonstrating that anti-CD32b mAb block binding of immune complexes to CD32b (17). In the bound conformation, the 6G08 F(ab) has an elbow angle, defined as the angle between the pseudo-dyad axes between the light and heavy chain variable and constant domains, of 136° (Fig. 1 *a*). To assess whether the F(ab) undergoes a conformational change upon binding, either through large-scale domain motions or more subtle changes at the interface, SAXS data were collected to investigate the conformation of the 6G08 F(ab) alone in solution.

Comparison of the theoretical scattering profile for the 6G08 F(ab) crystal structure, as calculated in CRYSOL, to the full experimental scattering data for the 6G08 F(ab) alone showed poor agreement, with a *χ*^2^ value of 5.81 (Fig. 1 *b*). The residual plot for the fit of the 6G08 F(ab) crystal structure to the full experimental data showed nonrandom features that appeared to worsen at a *q* range above 0.2 Å^−1^ (Fig. 1 *b*, *bottom panel*). Because of the increase in nonrandom features in the residual plots at *q* values >0.2 Å^−1^, we truncated the data to a maximal *q* value of 0.2 Å^−1^ in an attempt to avoid overfitting to the SAXS data. Refitting the 6G08 F(ab) crystal structure to a truncated SAXS data set with a maximal *q* value of 0.2 Å^−1^ resulted in an increased *χ*^2^ value of 13.95 (Fig. 1 *c*). This would initially suggest that the 6G08 F(ab) exhibits a distinct conformation in solution to that observed when bound to CD32b in the crystal structure.

To determine the structural differences between the free and bound forms of the F(ab), the conformational flexibility of the 6G08 F(ab) in solution was investigated through lengthy atomistic MD simulations.

### MD simulation of 6G08 analyzed with PCA

**Figure 2:**
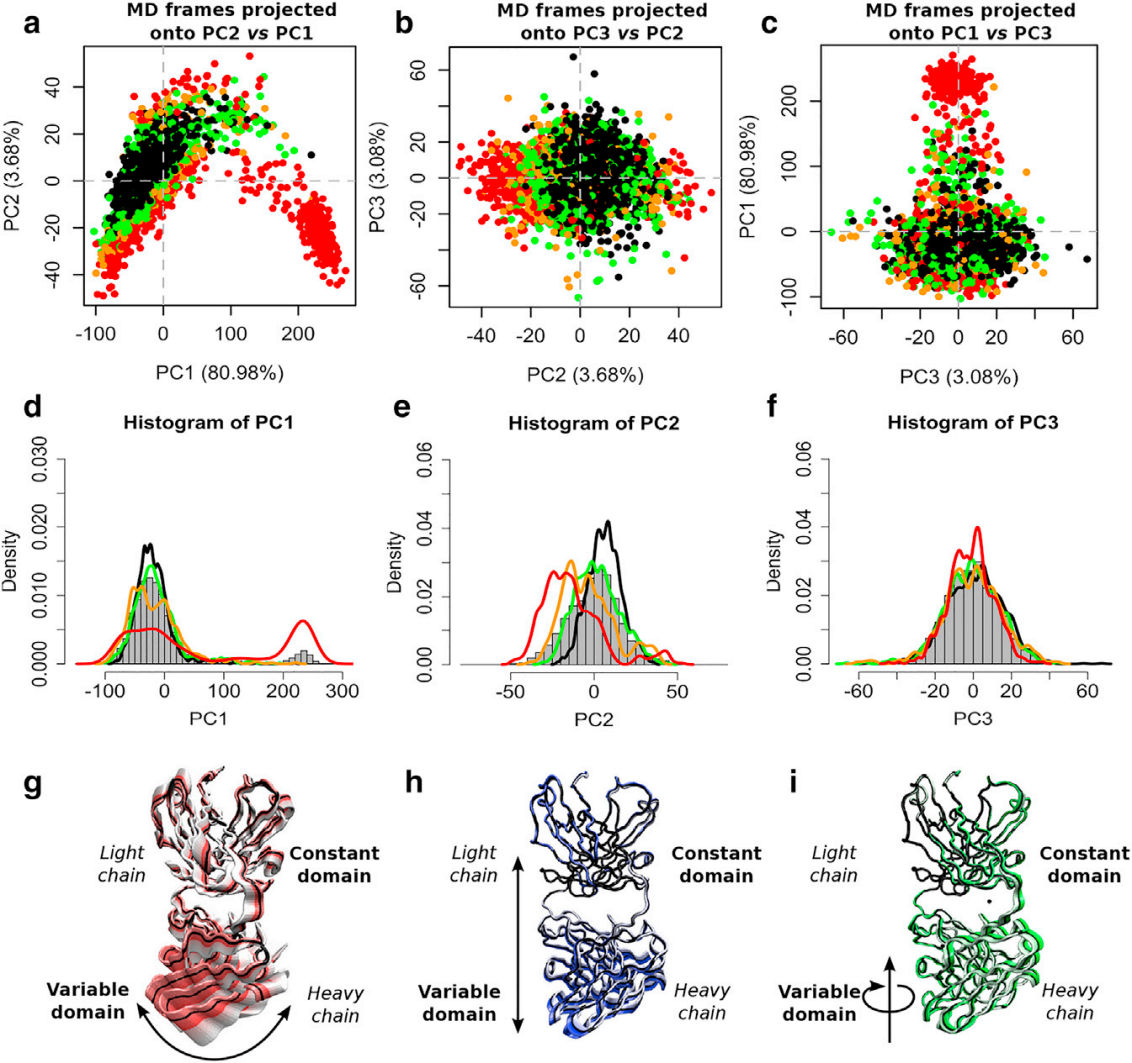
PCA of 6G08 F(ab) structures extracted from MD simulations identifies conformations with good agreement to SAXS data. (*a*–*c*) Structures extracted from MD simulations at 1 ns intervals are projected onto PC axes for the first three PCs. Color indicates *χ*^2^ fit to the truncated 6G08 F(ab) SAXS data, as detailed in the main text. (*d*–*f*) Histograms (*gray*) represent the total distribution of structures across the individual PC axes, and line plots of density distributions show structures in each *χ*^2^ fit category (*black*, *green*, *orange*, and *red*). Distributions are normalized to the individual population of each subset. (*g*–*i*) Motions captured by each PC; the C-terminal and N-terminal residues from the heavy and light chain, respectively, were removed for visualization. Arrows show general direction of motions and are not drawn to scale. Videos of these motions are available as Videos S1, S2, and S3. To see this figure in color, go online.

Three repeat 1 *μ*s MD simulations were conducted using the F(ab) structure isolated from the 6G08:CD32b crystal complex as a starting model. Structures extracted from the three simulations were analyzed via PCA to derive principal components (PCs). PCA of trajectory frames reveals a diverse range of visited conformations with distinct clusters of structures. A theoretical scattering curve for each frame extracted from the MD trajectory was calculated in CRYSOL and compared with the truncated 6G08 F(ab) SAXS data, and agreement between the scattering profiles was assessed by *χ*^2^ score. Each frame was then assigned a color based on *χ*^2^ value, as below. Fig. 2, *a*–*c* illustrates the distribution of structures across each two-dimensional combination of the first three PCs, which account for 87.7% of the total variance observed. The clustering profiles observed were found to be consistent when using multiple CRYSOL fitting parameters or the alternative scattering calculation program FoXS (Fig. S2).

Structures with good agreement to the 6G08 F(ab) SAXS data are observed to cluster at negative values of PC1 (Fig. 2, *a* and *c*, *black points*). To illustrate this clustering further, Fig. 2, *d*–*f* shows the total distribution of structures across PCs along with the normalized densities of structures exhibiting good (*χ*^2^ < 3.0, *black line*), fair (3.0 ≤ *χ*^2^ < 6.0, *green line*), poor (6.0 ≤ *χ*^2^ < 9.0, *orange line*), or very poor agreement (9.0 ≤ *χ*^2^, *red line*).

The motion captured in PC1 corresponds to a hinging motion of the two variable domains of the F(ab) about the flexible linker, resulting in a change in the F(ab) elbow angle (Fig. 2 *g*; Video S1). Structures projected at the extremes of the observed PC1 values, i.e., at −109 and +273 on the PC1 axis, have elbow angles of 126 and 233°, respectively. A clear separation is seen across PC1 between structures that show good agreement to the SAXS data and those that are poorly representative of the 6G08 F(ab) in solution (Fig. 2 *d*, *black* and *red lines*, respectively). This suggests that structures with an increased elbow angle (corresponding to large, positive values on the PC1 axis) between the F(ab) constant and variable domains are poorly descriptive of the SAXS data for the 6G08 F(ab). This agrees qualitatively with the fact that these structures were only rarely observed during the MD simulations. The total range of observed angles agrees well with previous measurements of angles in F(ab) with *λ* light chains in structures deposited in the PDB, which identified F(ab) elbow angles between 117 and 227° (46). The autocorrelation function of the F(ab) elbow angle for each trajectory is shown in Fig. S3, and the distribution of R_g_ values observed throughout the combined simulations is shown in Fig. S4.

Video S1. Motion Defined by Principal Component 1 (PC1)

In addition to PC1, structures are also partially separated according to their agreement to the SAXS data across PC2 (Fig. 2 *e*). PC2 represents an extension of the variable domains away from the constant region in a forward and backward motion (Fig. 2 *h*; Video S2). In contrast, PC3 does not discriminate well between structures with good or poor agreement to the SAXS data (Fig. 2 *f*) and represents the variable domains of the F(ab) twisting about the flexible linker (Fig. 2 *i*; Video S3). PC2 and PC3 each account for only 3–4% of the observed variance in the MD structures. Therefore, the major hinging motion between the constant and variable domains identified in PC1 is the key determinant of agreement between the atomic structures and experimental SAXS data for the 6G08 F(ab).

Video S2. Motion Defined by Principal Component 2 (PC2)

Video S3. Motion Defined by Principal Component 3 (PC3)

### Clustering of MD frames to generate representative structures

**Figure 3:**
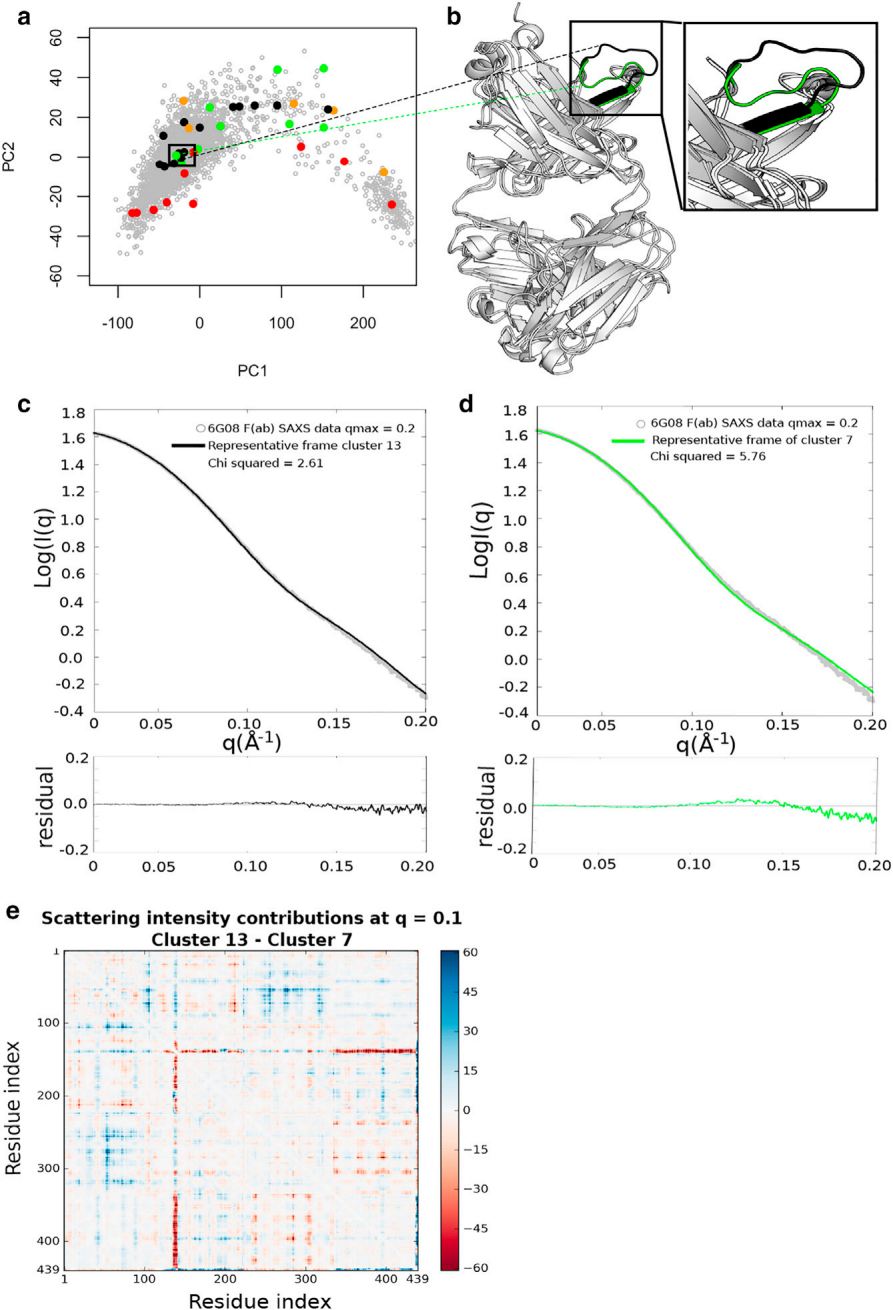
For a Figure360 author presentation of Fig. 3, see the figure legend at https://doi.org/10.1016/j.bpj.2018.03.040. Structures with similar PC values display different agreements to SAXS data depending on the conformation of a flexible loop in the F(ab) heavy chain. (*a*) All representative structures from hierarchical clustering of the MD trajectories are projected onto the original PC1 vs. PC2 axes. Colors indicate agreement of the representative structure to the truncated 6G08 F(ab) SAXS data, as previously described. Black box highlights clusters 7 (*green*) and 13 (*black*), which neighbor each other and are further analyzed in this figure. (*b*) Overlay of representative structures are shown for clusters 7 (*green highlight*) and 13 (*black highlight*). The major difference between structures is the position of a flexible loop, as shown in the inset. (*c* and *d*) Theoretical SAXS scattering profile of the representative frame from cluster 13 or cluster 7, respectively, compared to the truncated 6G08 F(ab) SAXS data. Bottom panels show the residual plots for the respective fits, with residuals defined as log I(q)_exp_ – log I(q)_mod_. *χ*^2^ scores are calculated in CRYSOL. (*e*) Difference matrices show the difference between residue contributions to scattering intensity between clusters 13 and 7 at a *q* value of 0.1 Å^−1^. Colored pixels represent a difference in residue contributions and are observed most clearly at residues 136–145, corresponding to the flexible loop identified in (*b*).To see this figure in color, go online. Figure360: An Author Presentation of Fig. 3.

Although PCA provides high-level discrimination between structures observed in the MD trajectories, finer structural differences between conformational populations can be evaluated with hierarchical clustering. This resulted in frames from the 3 × 1 *μ*s trajectories being sorted into 39 representative clusters (see Table S3 for details of each cluster). The representative structure of each individual cluster, taken as the trajectory frame with the lowest root mean-square displacement to the cluster center, was then projected onto the axes of the original PCA and colored according to *χ*^2^ value (Fig. 3 *a*).

The majority of representative structures project onto the same region of PC space as the previously identified productive region of structures (Fig. 2, *a*–*c*). Despite multiple clusters having similar PC values, the agreement of the scattering profile of the representative structure to the experimental 6G08 F(ab) SAXS data varies. In particular, the representative structures of clusters 7 and 13 project onto the same region of PC1, previously found to be the major motion responsible for determining *χ*^2^ values in the PCA, but they have *χ*^2^ values of 5.76 and 2.61, respectively (Fig. 3, *c* and *d*). We therefore chose to investigate the representative structures of these clusters further to determine the cause of *χ*^2^ variation within this region. The F(ab) elbow angle for the representative structure of the two clusters is also found to be similar, with elbow angles of 146 and 149° for clusters 7 and 13, respectively. Visual comparison of the representative structures for clusters 7 and 13 reveals that the major difference between the two structures is the conformation of a loop between residues 136 and 145 in the constant domain of the heavy chain (Fig. 3 *b*).

To validate the impact of the 136–145 loop position on the agreement of structures to the 6G08 F(ab) SAXS data, intensity difference matrices were calculated between the representative structures of clusters 7 and 13. As stated in the Materials and Methods, this involves the calculation of scattering intensity contributions from each residue-residue pair (defined by the distance *r*_*ij*_ between C*α* atoms and using residue-based form factors, *f*_*i,q*_, *f*_*j,q*_, in Eq. 2) for a given structure. Different conformations of the same protein have unique sets of inter-residue distances and therefore unique sets of scattering intensities due to these pairwise distances. Subtraction of these pairwise scattering intensity contributions then allows the differences in residue-by-residue scattering from each conformation (or cluster, as described here) to be visualized, highlighting the key areas where conformational differences invoke large changes in the predicted SAXS scattering intensity. A representative matrix at scattering vector *q* = 0.1 Å^−1^ is shown in Fig. 3 *e*. Further difference matrices are available in Fig. S5.

At low *q* values (≤0.1Å^−1^), the difference matrices highlight that the contribution of residues 136–145 to the overall scattering intensity varies greatly between the two structures. At larger scattering vectors, the intensity differences are slightly more evenly distributed across all residues, as may be expected in this low signal region. The conformation of the F(ab) heavy chain loop is therefore thought to influence the agreement between the theoretical and experimental curves at a *q* range of <0.1 Å^−1^. This can be seen in the residual plots for fits presented in Fig. 3, *c* and *d*, which show improved agreement for the representative structure of cluster 13 between *q* = 0.05 and 0.15 Å^−1^ (Fig. 3 *c*, *bottom panel*). The difference matrices and residual plots therefore confirm that the crucial differences between the representative structures of clusters 7 and 13 are centered around residues 136–145, as previously observed from visual inspection (Fig. 3 *b*). The representative structure of cluster 13, which shows improved agreement to the 6G08 F(ab) SAXS data, has an extended loop conformation at these residues (Fig. 3, *b* and *c*, *inset*).

**Figure 4:**
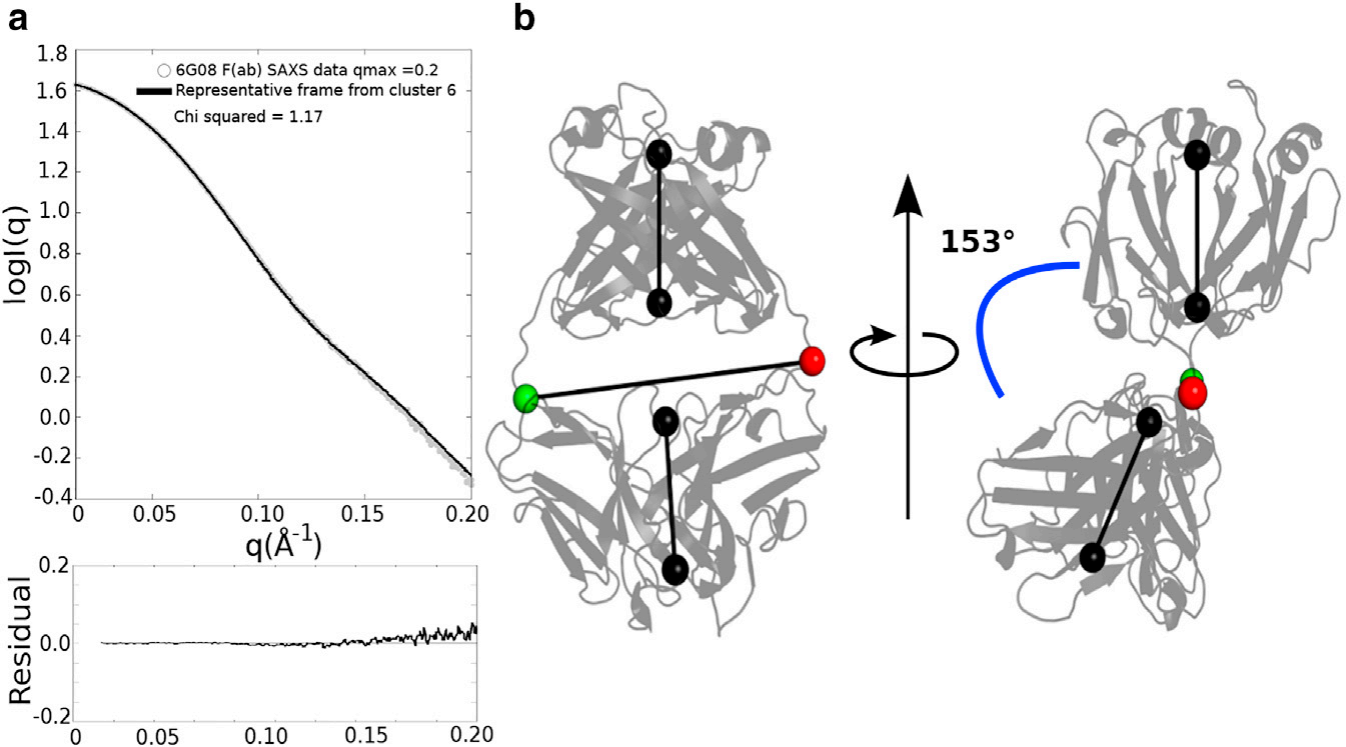
Cluster 6 shows best agreement with the 6G08 F(ab) SAXS data. (*a*) Comparison of cluster 6 (*black*) to the truncated SAXS data for the 6G08 F(ab) (*gray*) is shown. The model shows good agreement to the data with a *χ*^2^ score of 1.17. Bottom panel shows the residual plots for the respective fit; residuals are defined as log I(q)_exp_ – log I(q)_mod._*χ*^2^ scores are calculated in CRYSOL. (*b*) Structure of the representative frame for cluster 6 (frame 2065), which has an F(ab) elbow angle of 153°, is shown. Second structure represents cluster 6 rotated to better show the elbow angle between F(ab) domains. To see this figure in color, go online.

Cluster 6 shows the best agreement to the 6G08 F(ab) SAXS data. The representative structure for this cluster has a *χ*^2^ score of 1.17 and overlays well with the experimental SAXS profile for the 6G08 F(ab) at *q* values of <0.2 Å^−1^ (Fig. 4 *a*). As previously observed in cluster 13, the 136–145 heavy chain loop is in an extended conformation, whereas the overall F(ab) elbow angle is 153° (Fig. 4 *b*).

The single representative structure of cluster 6 describes the SAXS data well up to a *q* value of 0.2 Å^−1^. Because of the inherent flexibility of the F(ab) structure as identified in the MD simulations, it is likely that an ensemble approach would be required to explain the SAXS data to a higher *q* value. Preliminary ensemble modeling refinements showed good agreement to the SAXS data up to a *q* value of 0.35 Å^−1^. As seen in the fitting of single structures to the SAXS data, all structures chosen for the optimal ensembles contained an open loop conformation (Fig. S7).

### Comparison of 6G08 F(ab) crystal structure with MD simulation

**Figure 5:**
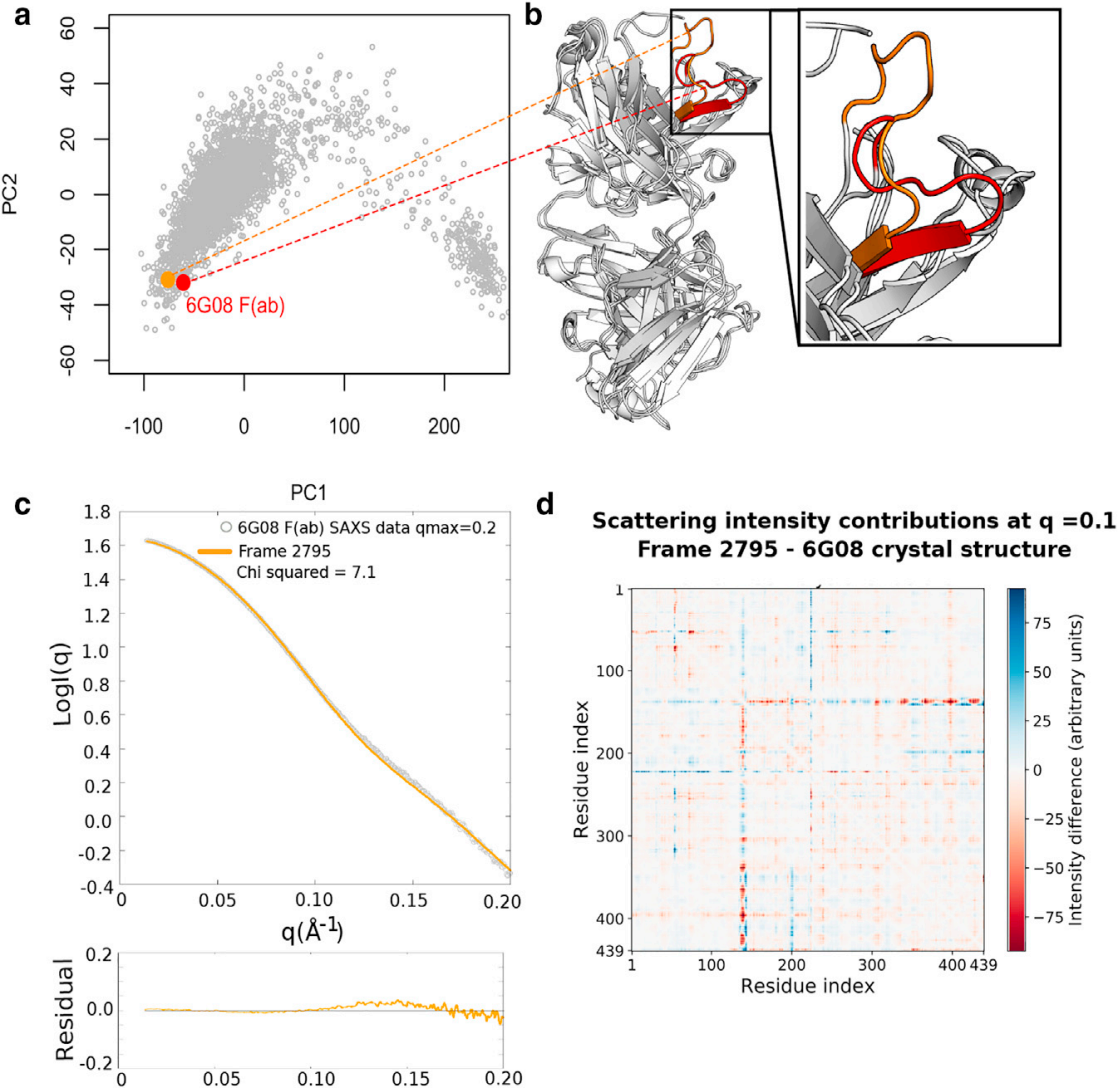
Conformation of the flexible loop at residues 136–145 in the F(ab) heavy chain is responsible for the difference between 6G08 F(ab) crystal structure and SAXS data. (*a*) 6G08 F(ab) crystal structure (*red*) is projected onto the PC1 and PC2 axes from the original PCA of frames extracted from MD simulations. A model with similar PC values shows improved agreement with the 6G08 F(ab) SAXS data (frame 2795, *orange*). (*b*) Bound form of the 6G08 F(ab) is shown isolated from the crystal complex aligned to frame 2795. The major difference between structures is the conformation of the loop between residues 136 and 145 in the F(ab) heavy chain, shown in the inset. (*c*) Theoretical SAXS scattering profile of frame 2795 (*χ*^2^ 7.1) is shown compared to the truncated 6G08 F(ab) SAXS data. Bottom panel shows the residual plot for the respective fit; residuals are defined as log I(q)_exp_ – log I(q)_mod._*χ*^2^ scores are calculated in CRYSOL. (*d*) Difference matrix between the 6G08 F(ab) crystal structure and frame 2795 at a *q* value of 0.1 Å^−1^. Colored pixels represent a difference in residue contributions and are again observed at residues 136–145. To see this figure in color, go online.

The analysis of the MD trajectories above shows that overall agreement with the 6G08 F(ab) solution SAXS data may be affected by both large domain motions in the F(ab) elbow angle and small loop fluctuations. The structure of the F(ab) as observed in the CD32b crystal complex was therefore similarly compared to the MD simulations to investigate the reason for its high *χ*^2^ score of 13.95 when compared to the truncated SAXS data with a maximal *q* value of 0.2 Å^−1^ (Fig. 1 *c*). Fig. 5 *a* shows the 6G08 F(ab) crystal structure projected onto the PC1 vs. PC2 axes, which places the F(ab) crystal structure onto the left-hand region of PC1, similar to structures that had good agreement to the 6G08 F(ab) SAXS data. This would suggest that there is not a large conformational change in the F(ab) elbow angle between the free and bound forms of the F(ab).

As the conformation of the 136–145 loop had been found to influence the agreement of structures in this region with the SAXS data, the conformation of this loop in our crystal was compared and found to be in a “bent” conformation similar to that of cluster 7 (Figs. 3 *b* and 5 *b*, respectively). The crystal structure was also compared to a trajectory frame from the same region of the PCA with improved agreement to the SAXS data, i.e., frame 2795 (*χ*^2^ 7.1, Fig. 5 *c*). Visual comparison confirmed that the major difference between these structures was the conformation of the 136–145 loop, with frame 2795 having an extended loop conformation as observed in the well-fitting cluster 13. Additionally, frame 2795 and the 6G08 F(ab) crystal structure have similar elbow angles of 127 and 134°, respectively. The calculation of the SAXS difference intensity matrices confirmed that the major differences between the 6G08 F(ab) crystal structure and frame 2795 at low *q* vectors were centered around residues 136–145, as previously observed for the representatives of clusters 7 and 13 (Figs. 3 *e* and 5 *d*, respectively; additional difference matrices are shown in Figs. S5 and S6). When looking at the residual plots, frame 2795 shows improved agreement to the SAXS data between *q* = 0.05 and 0.15 Å^−1^, as previously seen for cluster 13 (Figs. 3 *c* and 5 *c*, respectively). This would suggest that the “bent” and “extended” conformations of the 136–145 loop in the 6G08 F(ab) structures are also responsible for determining the agreement of the atomistic models to the SAXS data for the 6G08 F(ab) alone in solution. This again points to an absence of a large conformational change between bound and unbound F(ab) and the presence of more subtle fluctuations that can be probed well by the timescales accessible to atomistic MD.

## Discussion

In this study, we determined the structure of the F(ab) domain of the agonist mAb 6G08 in complex with the extracellular region of CD32b and assessed differences between free and bound forms of the F(ab) using complementary biophysical and theoretical techniques to study the structure and dynamics of the 6G08 F(ab).

Previous structural investigations of F(ab) domains have identified variability in the angle between the F(ab) constant and variable domains, referred to as the elbow angle (46, 47, 48). Mutations within the region linking the F(ab) constant and variable domains have been found to influence the elbow angle with potential to alter binding functions and protein dynamics (48). The conformation of the 6G08 F(ab) is therefore likely to influence interactions with CD32b and consequently could determine the activity of the mAb.

In this study, we perform extensive MD simulations to investigate the dynamics of the 6G08 F(ab) on the microsecond timescale. Our simulations also identify hinging of the elbow angle to be a key conformational motion of the F(ab), as previously described, and indicate that this is one of the key determinants of agreement with SAXS data (Fig. 2 *d*). However, comparison of the bound crystal structure of 6G08 F(ab) to structures extracted from MD, which showed good agreement with the SAXS data for the 6G08 F(ab) alone, suggests that although changes in elbow angle may occur (134–153° for the crystal structure and the best-fitting MD cluster 6, respectively), there are no large domain reorientations upon binding of the 6G08 F(ab) to CD32b.

By color coding the PCA according to the agreement of the structure to experimental SAXS data, our method allowed the identification of small, localized structural differences that also influence the agreement of the theoretical and experimental scattering profiles up to a resolution of 0.2 Å^−1^. The loop between residues 136–145 of the F(ab) heavy chain appears to make a contribution to the differences in observed *χ*^2^ fit to SAXS data for the 6G08 F(ab) alone. This is true both between the simulation frames themselves and between the simulation and experimental crystal structure.

The identified 136–145 heavy chain loop from our simulations lies within the constant domain of the F(ab), which is not part of the antibody CDR region and does not contact the CD32b receptor. When identifying the position of this heavy chain loop in the initial 6G08:CD32b complex, it is evident that this loop is in close proximity to other symmetry mates within the crystal lattice (Fig. S8). Therefore, it is possible that the crystal packing conditions may have influenced the conformation of the 136–145 heavy chain loop in the crystal structure of the F(ab). This further re-enforces the advantages of acquiring complementary solution-phase data when interpreting protein conformations in crystal structures.

Although solution-phase data such as SAXS can complement x-ray crystallography data, comparison of low-resolution bead structures and rigid body approaches alone are unlikely to allow identification of small, localized conformational changes that may have an impact on protein function. It is only through extensive analysis of MD simulation data that localized motions such as the 136–145 heavy chain loop conformation could be identified. MD methods hold numerous advantages over other model generation techniques. Most notably, they can use completely atomistic environments (for both biomolecule and solvent), the interactions are calculated using classical physical principles (meaning that generated structures are more likely to be physically relevant), and the dynamics can be explored over lengthy timescales (up to microseconds). Even these lengthy microsecond timescales of dynamics may not be sufficient to converge results with conventional MD so that identical solution ensembles are observed between independent repeat simulations. Promising enhanced sampling methodologies have recently been developed that restrain MD simulations to help recreate an experimental solution ensemble (44), but application of these methods to large biomolecular systems with real experimental data remains limited at this time.

Our current method, a combination of MD, x-ray crystallography, and SAXS data, identifies single F(ab) conformations that explain the experimental SAXS well to a resolution of 0.2 Å^−1^. Because of the dynamic nature of proteins in solution, it is likely that an ensemble approach may be required to fit to higher resolutions of *q* (Fig. S7). Historically, the low resolution of SAXS data has made the identification of the underlying protein ensemble challenging. However, new approaches using MD to improve the calculation of accurate scattering profiles from proteins in solution have been shown to be useful tools in accurately predicting the solution ensemble of biomolecules, further emphasizing the synergistic combination of theoretical simulation and SAXS data (49, 50, 51). Overall, we believe that the complementary use of theoretical simulation and experimental data can add additional insight and value in the determination of protein conformation and dynamics.

## Conclusions

Using an antibody F(ab) fragment, we demonstrate that MD combined with PCA can be used to understand structural differences between solution phase SAXS and crystallographic data. By incorporating the agreement of each individual MD structure with our experimental SAXS data into the PCA, we were able to identify both global and localized motions important for fitting atomic coordinates to the solution phase data. We show that F(ab) elbow angle and additional changes in localized loop regions were important when fitting atomic MD structures to the SAXS data and that these localized loop regions were responsible for the poor agreement of our crystallographic structure to solution phase SAXS data for the F(ab). We believe this method will be generally applicable to the study of additional macromolecular systems and complexes.

## Author Contributions

E.J.S. and R.T.B. designed the research, performed the research, analyzed the data, and wrote the article. C.M.O. analyzed the data and wrote the article. B.F., G.L., and I. Teige contributed research materials. M.S.C., I. Tews, and J.W.E. designed the research and wrote the article.

## Supporting Citations

References (52, 53, 54, 55, 56, 57, 58, 59, 60) appear in the Supporting Material.

## Supporting Material

Document S1. Supporting Materials and Methods, Figs. S1–S8, and Tables S1–S3

Document S2. Article plus Supporting Material
