# Supplementary material for: Evaluating Anti-CD32b F(ab) Conformation Using Molecular Dynamics and Small-Angle X-Ray Scattering
Source: Biophys J. 2018 Jul 17;115(2):289–99. doi: 10.1016/j.bpj.2018.03.040 (PMC6050753; doi:10.1016/j.bpj.2018.03.040)
Supplement: Document S1. Supporting Materials and Methods, Figs. S1–S8, and Tables S1–S3 [file mmc1.pdf]

Biophysical Journal, Volume *115*

Supplemental Information

Evaluating Anti-CD32b F(ab) Conformation Using Molecular Dynamics and Small-Angle X-Ray Scattering

Emma J. Sutton, Richard T. Bradshaw, Christian M. Orr, Bjorn Frendéus, Gunilla Larsson, Ingrid Teige, Mark S. Cragg, Ivo Tews, and Jonathan W. Essex

Supporting Figures:

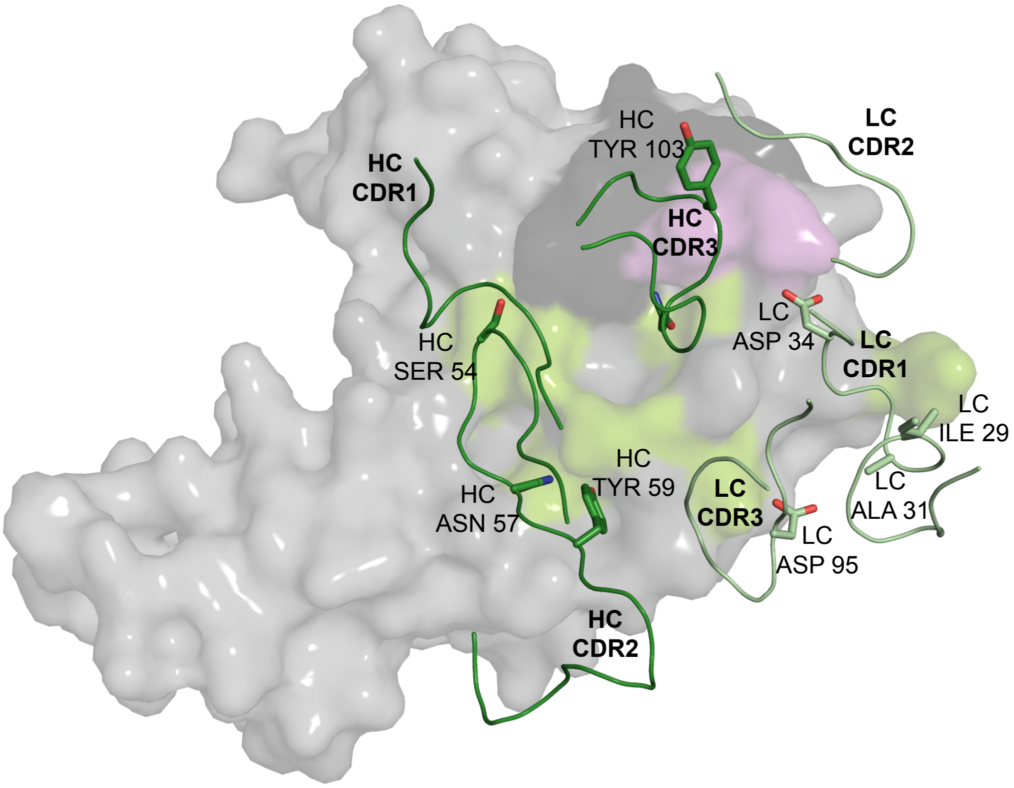

Figure S1 – Interacting residues in the 6G08 F(ab):CD32b crystal complex. CD32b is represented as grey surface, residues interacting with the 6G08 F(ab) complementarity determining region loops (CDR, green ribbons) are shown in green and residues identified to be part of the IgG Fc binding region as defined in (1) shown in black shading on the surface. Residues involved in 6G08 F(ab) interactions that are also part of the IgG Fc binding region are shown in pink. Only the CDR regions of the 6G08 F(ab) are shown with the heavy (HC) in dark green and light chain (LC) in light green. Residues represented as sticks are identified to be involved in interactions with CD32b as identified by the PDBePISA server.

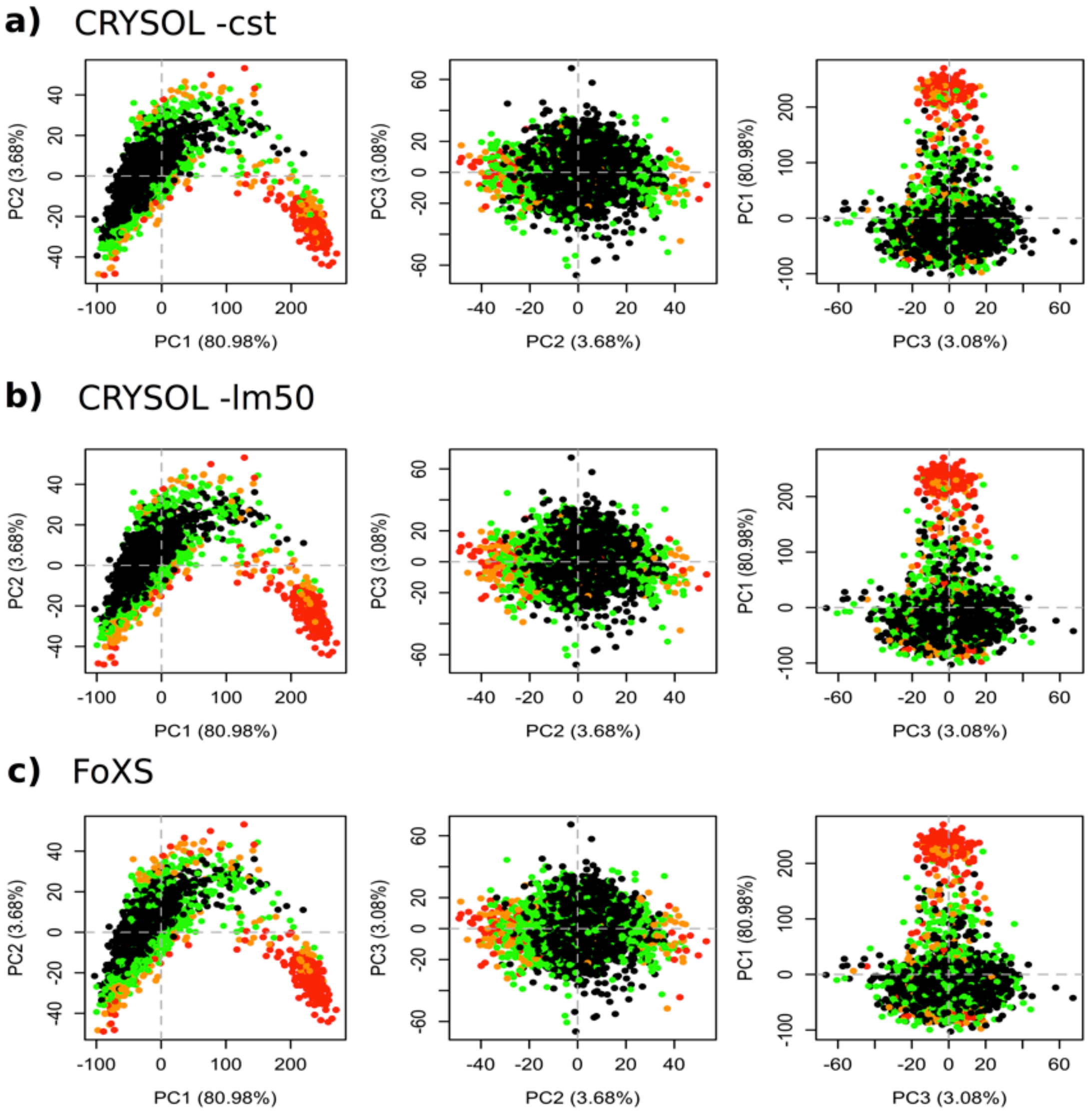

Figure S2 – Validation of CRYSOL fitting. Additional SAXS fitting parameters were used to ensure the trends observed in Figure 2 of the main text were independent of the fitting procedure. Structures extracted from MD simulations at 1 ns intervals were projected onto PC axes for the first 3 PCs. Colour indicates χ^2 fit to the full 6G08 F(ab) SAXS data, a) shows the χ^2 fit when fitting to the full range of SAXS data using the additional option for constant subtraction in CRYSOL b) shows χ^2 fit when using an increased number of spherical harmonics in CRYSOL (50 in this case), and c) shows χ^2 fit when using the alternative scattering calculations program, FoXS. No obvious differences are observed between the different results using the fitting parameters.

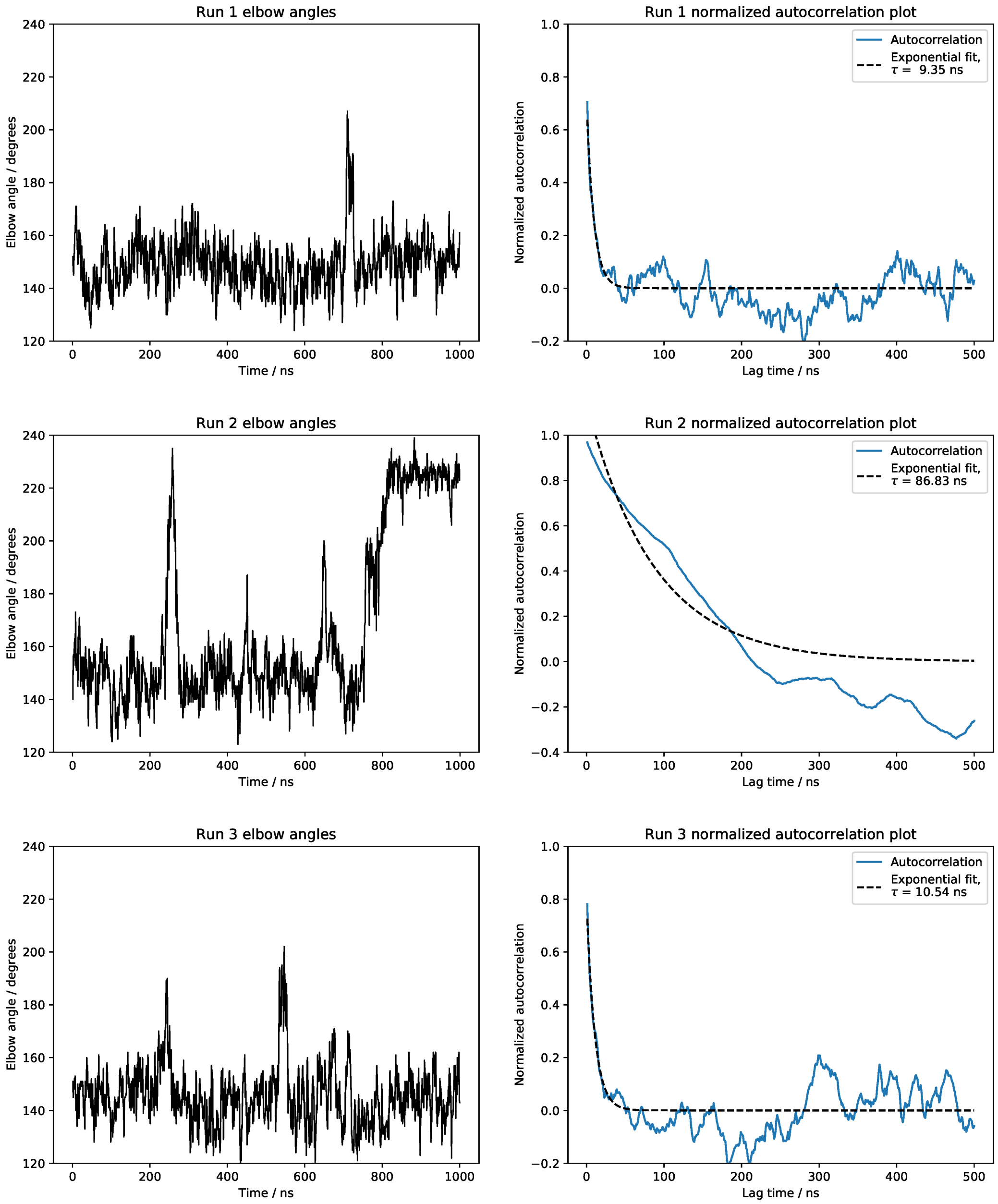

Figure S3 – F(ab) elbow angles and normalized autocorrelation functions of elbow angle for each of the three independent simulations. Autocorrelation functions were calculated as:

$$C_t = \frac{\langle A_0 A_t \rangle}{\langle A_0 A_0 \rangle} \tag{S1}$$

where A is the value $(\theta - \bar{\theta})$, i.e. the deviation of the elbow angle from the mean. Owing to the lack of periodicity in the elbow angle calcuation, elbow angles in degrees were used directly rather than a cosine function of the elbow angle. Autocorrelations were evaluated at lag times, t, of up to 500 ns and averaged over every starting frame of the trajectory. Correlation times, τ, were estimated using a single exponential decay fit to the underlying function. Runs 1 & 3 show fast dynamics and short correlation times of ~10 ns, while Run 2 shows elbow angle dynamics across multiple timescales. We do not attempt to estimate multiple correlation times for Run 2 as slow conformational dynamics are unlikely to be converged during the timescale of our simulations.

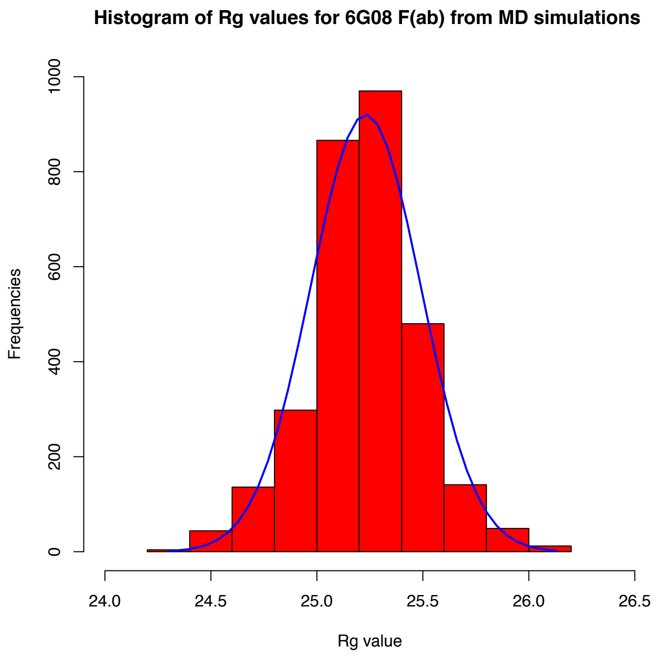

Figure S4 – Histogram of the radius of gyration (Rg) values for the 6G08 F(ab) structures extracted from all three molecular dynamics simulations. Rg values for each structure were calculated as part of the fitting procedure in CRYSOL (2). Red bars represent the frequency of the Rg values observed and the blue line shows the normal distribution fitted using the mean and standard deviation of the Rg values. The majority of structures had an Rg value between 25.0 and 25.5 Å which is close to the experimental Rg value of 25.5 Å.

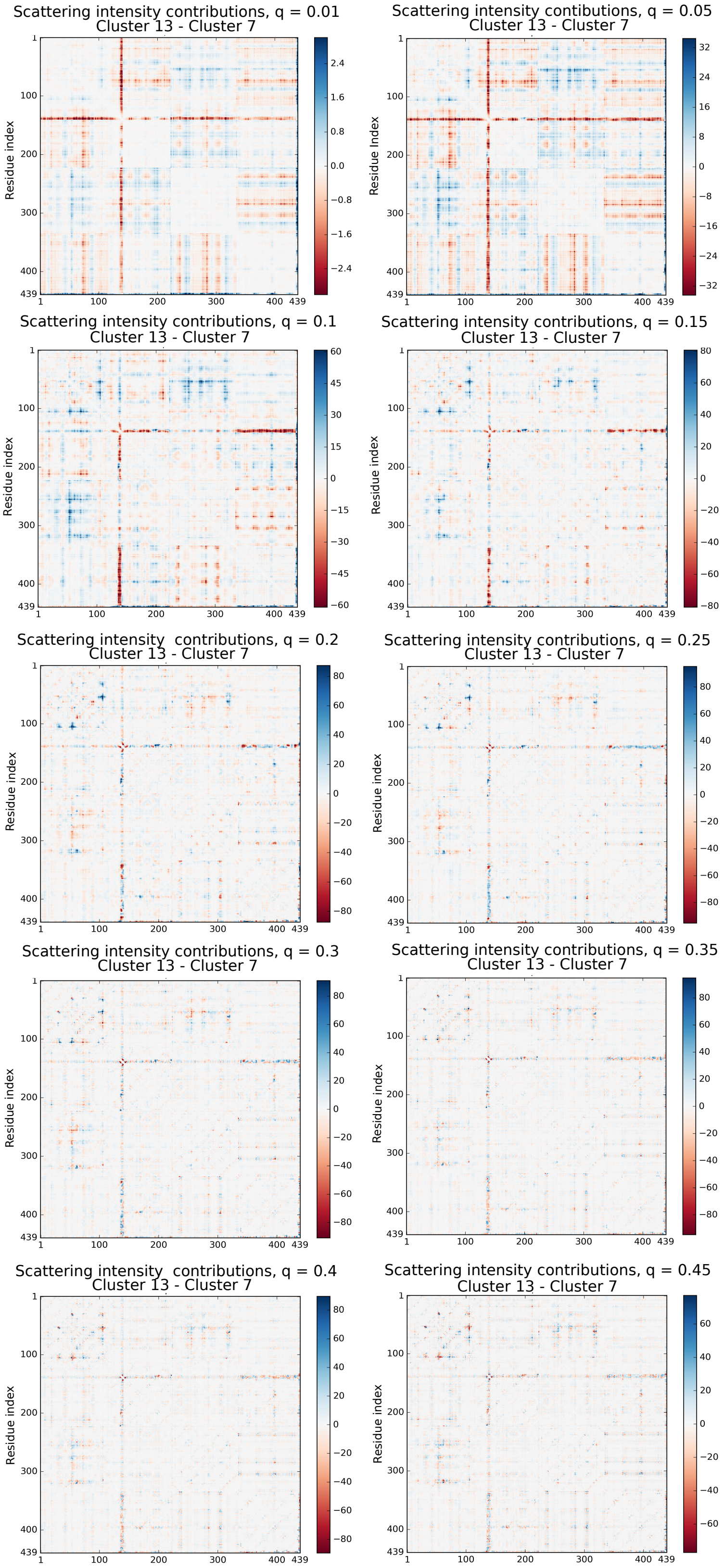

Figure S5 – Intensity difference matrices showing the difference in scattering intensity contributions between the representative structures of clusters 7 and 13 at multiple q values (Å^{-1}). Coloured pixels represent a difference in residue contributions and are often observed at residues 136-145.

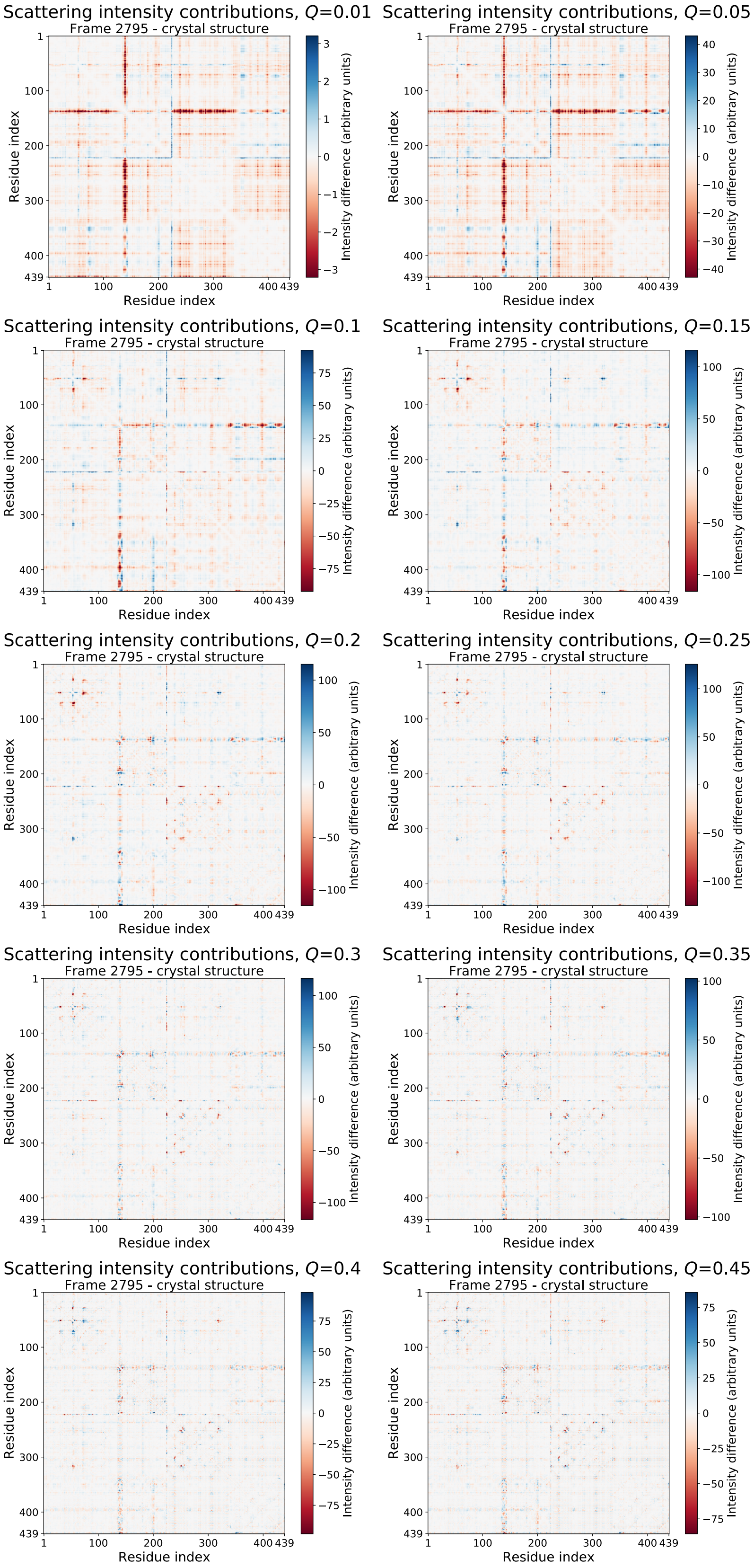

Figure S6 – Intensity difference matrices showing the difference in scattering intensity contributions between frame 2795 and the crystal structure of the 6G08 F(ab) at a multiple q values (Å^{-1}). Coloured pixels represent a difference in residue interactions and are often observed at residues 136-145.

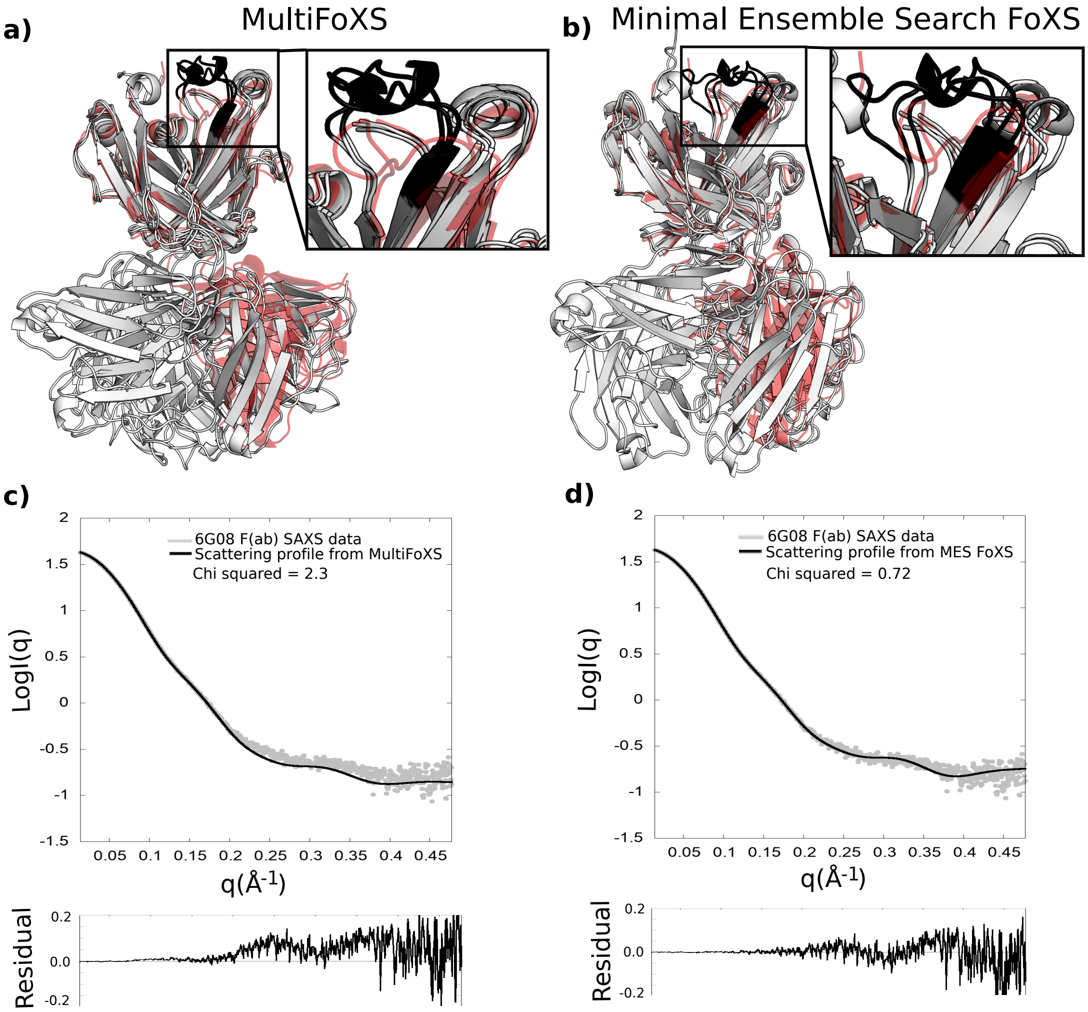

Figure S7 – Preliminary ensemble modelling shows models in the optimal ensemble have open loop conformations. a) Ensemble modelling using the MutliFoXS online server. b) Ensemble modelling using the Minimal Ensemble Search fit in FoXS. White structures represent the conformation of models in the optimal ensemble with the 136-145 loop highlighted in black. The initial 6G08 F(ab) structure from the crystal complex is shown in red with the bent loop conformation. Structures are aligned on the F(ab) constant domain as described in the main text c) and d) show the comparison of the theoretical scattering profiles for the ensembles shown in a) and b), respectively (black), to the 6G08 F(ab) SAXS data (grey). Residuals for the fits are shown below.

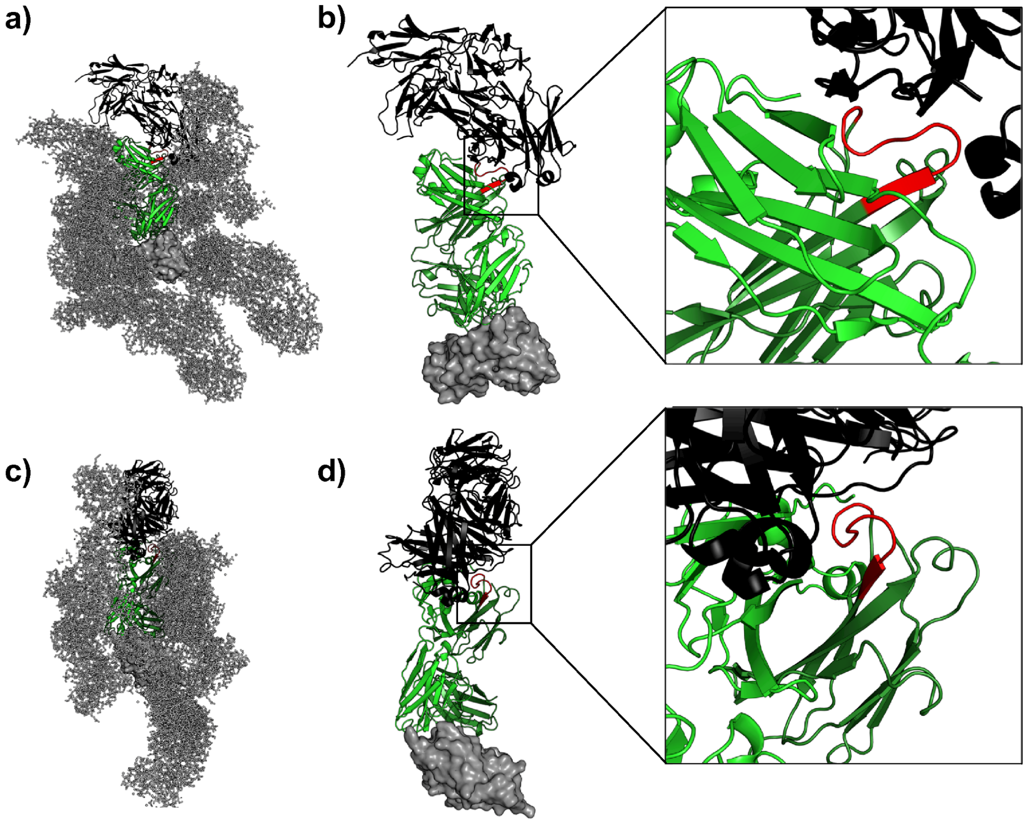

Figure S8 – Symmetry mates in the 6G08:CD32b crystal structure may have influenced the conformation of the 136 to 145 loop in the F(ab) heavy chain. a) The initial structure of the 6G08 F(ab) (green) in complex with CD32b (grey surface) with symmetry mates shown as grey sticks and a possible interacting structure shown in black. b) The 6G08 F(ab) (green) in complex with CD32b (grey surface) shown with symmetry mate in close contact to the 136 to 145 loop region (red) in the F(ab) heavy chain. Inset shows the proximity of the symmetry mate to the 136 - 145 loop. c) and d) represent a) and b) rotated by 90°.

Supporting Tables:

Table S1 – Data collection and refinement statistics for the 6G08 F(ab): CD32b crystal structure

	6G08 F(ab):CD23b
**Data collection**	
Space group	P 31 2 1
Cell dimensions	
a, *b*, *c* (Å)	158.73 158.75 93.62
α, β, γ (°)	90 90 120
Resolution (Å)	45.83-2.99 (3.10- 2.99)
R_{sym} or R_{merge}	0.03109 (0.2885)
$I / \sigma I$	13.89 (2.11)
Completeness (%)	99.63 (96.91)
Redundancy	2.0 (2.0)
**Refinement**	
Resolution (Å)	2.99
No. reflections	55286 (5264)
R_{work} / R_{free}	0.1941/0.2310
No. atoms	
Protein	4063
Ligand/ion	-
Water	21
B-factors	
Protein	87.43
Ligand/ion	-
Water	77.41
R.m.s. deviations	
Bond lengths (Å)	0.018
Bond angles (°)	2.14

*Values in parentheses are for highest-resolution shell.

Table S2 – Fitting parameters for CRYSOL scattering calculations presented in the main paper

CRYSOL fitting parameter	Value
Maximum q value ($Å^{-1}$)	0.2
Vol ($Å^{3}$)	52303 - 60785
Ra (Å)	1.4 – 1.8
Dro (e $Å^{-3}$)	0.0 – 0.075
Electron density of bulk solvent (e/$Å^{3}$)	0.334
Electron density of the hydration layer (Hydra) (e/$Å^{3}$)	0.316 – 0.337

Table S3 – Details of representative frames for clusters extracted from combined 3 μs MD trajectory of the 6G08 F(ab) fragment

Cluster	Number of frames in cluster	Representative frame	χ^2 of cluster representative (CRYSOL – qmax 0.2 Å^{-1})	χ^2 of cluster representative (CRYSOL – full dataset)	χ^2 of cluster representative (FoXS)	Rg of representative frame (Å)	Elbow angle of representative frame (degrees)
1	991	280	3.58	1.96	1.69	25.14	145
2	519	28	2.47	1.71	2.07	25.24	137
3	307	2783	1.55	1.16	1.74	25.32	141
4	193	1864	42.48	15.32	18.15	24.60	226
5	179	921	5.01	2.69	2.4	25.15	153
6	151	2065	1.17	1.10	1.49	25.46	153
7	87	1127	5.76	2.97	3.03	25.16	146
8	81	1446	2.75	2.12	2.10	25.36	166
9	63	2834	0.72	0.71	1.80	25.50	153
10	57	1573	5.16	2.76	2.16	25.12	155
11	50	2209	2.91	1.83	1.49	25.21	147
12	40	808	3.40	1.95	1.77	25.21	154
13	39	334	2.61	1.49	1.46	25.20	149
14	30	1770	6.67	4.28	4.97	25.46	191
15	29	2545	2.34	1.92	2.43	25.41	170
16	25	1788	11.51	4.92	5.24	24.96	197
17	20	1010	2.10	1.26	1.90	25.31	151
18	19	718	1.55	1.45	1.85	25.48	188
19	19	1389	6.72	3.85	4.75	25.98	149
20	17	1113	12.46	5.27	5.66	25.05	130
21	15	1254	6.51	3.89	4.28	25.51	209
22	10	2661	10.42	4.54	4.20	24.92	147
23	9	2017	9.24	3.52	4.80	25.02	137
24	8	1016	5.19	3.21	3.72	25.27	163
25	5	397	1.21	1.17	1.46	25.47	151
26	5	2133	14.06	5.47	6.97	25.07	130
27	5	1257	7.54	3.03	3.35	25.16	224
28	4	10	1.68	1.16	1.99	25.35	171
29	4	2244	5.33	3.84	3.92	25.34	189
30	3	1181	3.48	2.74	2.86	25.44	156
31	3	710	5.06	3.13	3.80	25.82	200
32	3	1249	13.97	7.04	7.78	25.97	189
33	2	1003	10.97	4.72	5.34	25.00	157
34	2	1179	0.80	1.09	1.56	25.58	143
35	2	2547	1.83	1.56	2.10	25.43	202
36	1	1726	6.48	3.88	4.58	25.65	147
37	1	1852	13.4	5.28	5.90	24.96	206
38	1	2009	13.98	5.22	6.81	25.03	140
39	1	2409	15.77	6.00	6.66	24.85	147

Supporting text:

Text S1 - Molecular dynamics simulation protocol

All three repeat simulations were carried out using an identical protocol as follows. Simulations were performed using the *PMEMD* and *PMEMD.cuda* modules of the Amber 16 software suite (3). Systems were first energy minimised for a total of 2500 steps, of which the first 1000 steps used a steepest descent protocol and the latter 1500 steps used a conjugate gradient protocol. Systems were heated to 300 K over 50 ps under NVT conditions, followed by 100 ps of equilibration at 1 bar under NPT conditions. Following equilibration each system was then simulated for 1 μs under NPT conditions. Structures were extracted from the simulations at 1 ns intervals, giving a total of 1000 snapshots for analysis from each simulation.

All simulations were performed under periodic boundary conditions using an 8 Å direct space cut-off for nonbonded interactions, with an analytical dispersion correction used to account for long-range van der Waals interactions and a Particle Mesh Ewald summation for long-range electrostatics (4). All simulations used a 2 fs time step with SHAKE (5) to constrain all bonds involving hydrogen. A Langevin thermostat (6–8) was used to maintain temperature in all simulations, with a collision frequency of 2 ps^{-1}. A Berendsen barostat (9) with pressure relaxation time of 2.0 ps was used to maintain pressure during system equilibration to 1 bar whereas a Monte Carlo barostat was used throughout the 1 μs production simulations, with 100 steps between volume change attempts.

Text S2 – SAXS scattering calculation and fitting procedure

CRYSOL was used to calculate a scattering intensity profile, and fit, to the experimental SAXS data for each 6G08 F(ab) computational structure. CRYSOL uses spherical harmonics with a multipole expansion to compute theoretical SAXS profiles from atomic co-ordinates based on the equations in (2). CRYSOL fits the theoretical scattering curve to experimental data by minimizing the discrepancy according to a chi square value. The chi square value is calculated as:

$$\mathrm{X}^2(r_0\delta\rho) = \frac{1}{N_p}\sum_{i=1}^{N_p}\left[\frac{I_e(s_i) - cI(s_i, r_0, \delta\rho)}{\sigma(s_i)}\right]^2 \qquad \text{(S2)}$$

Where I_e is the experimental scattering profile, I is the calculated scattering profile from a set of atomic co-ordinates, r_0 is the atomic radius, $\delta\rho$ the hydration shell, $\sigma(s_i)$ the experimental error for each data point (s_i), N_p the number of experimental points and c is a scaling factor (2). The scaling factor c includes three parameters used to minimize discrepancy between experimental and theoretical scattering profiles, the three parameters which are optimized include; 1) the average displaced solvent volume per atomic group, 2) the contrast of the hydration shell and 3) the relative background (2). By default, CRYSOL will find the optimal hydration shell contrast, excluded volume and atomic group radius separately for each structure to optimize fitting to the experimental data. The range of values used in the scattering calculations presented in the main text are shown in Table S2 of the supporting material. All fits presented in the main text were calculated using CRYSOL with default options and fitted to a truncated SAXS data set with a maximum q value of 0.2 A^{-1}.

To ensure our results were not influenced by the fitting procedure used, further CRYSOL fitting was also conducted using the options shown in figure 2 of the supporting material. This included fitting the

structures to the full range of experimental SAXS data in CRYSOL using the constant subtraction option to take in to account potential errors associated with buffer subtraction (figure 2a) or using an increased order of harmonics (50, figure 2b). In addition, the alternative scattering calculation program FoXS (10, 11), was also used to validate the CRYSOL fitting. FoXS scattering calculations were run using the online server available at: https://modbase.compbio.ucsf.edu/foxs/, using default parameters, data shown in figure S2c of the supporting material. Preliminary ensemble modelling was conducted automatically using MultiFoXS (12) and Minimal Ensemble Search (13) using default parameters as part of FoXS online server and used the 39 structures that represent the clusters described in Table S3.

Supporting references:

1. Veri, M.C., S. Gorlatov, H. Li, S. Burke, S. Johnson, J. Stavenhagen, K.E. Stein, E. Bonvini, and S. Koenig. 2007. Monoclonal antibodies capable of discriminating the human inhibitory Fcγ-receptor IIB (CD32B) from the activating Fcγ-receptor IIA (CD32A): Biochemical, biological and functional characterization. Immunology. 121: 392–404.

2. Svergun, D., C. Barberato, and M.H. Koch. 1995. CRYSOL - A program to evaluate X-ray solution scattering of biological macromolecules from atomic coordinates. J. Appl. Crystallogr. 28: 768–773.

3. Case, D.A., R.M. Betz, D.S. Cerutti, T.E. Cheatham, T.A. Darden, R.E. Duke, T.J. Giese, H. Gohlke, A.W. Goetz, N. Homeyer, S. Izadi, P. Janowski, J. Kaus, A. Kovalenko, T.S. Lee, S. LeGrand, P. Li, C. Lin, T. Luchko, R. Luo, B. Madej, D. Mermelstein, K.M. Merz, G. Monard, H. Nguyen, H.T. Nguyen, I. Omelyan, A. Onufriev, D.R. Roe, A. Roitberg, C. Sagui, C.L. Simmerling, W.M. Botello-Smith, J. Swails, R.C. Walker, J. Wang, R.M. Wolf, X. Wu, L. Xiao, and P.A. Kollman. 2016. AMBER 16. University of California, San Francisco.

4. Darden, T., D. York, and L. Pedersen. 1993. Particle mesh Ewald: An N log(N) method for Ewald sums in large systems. J. Chem. Phys. 98: 10089.

5. Ryckaert, J.P., G. Ciccotti, and H.J.C. Berendsen. 1977. Numerical-Integration of Cartesian Equations of Motion of a System with Constraints - Molecular-Dynamics of N-Alkanes. J. Comput. Phys. 23: 327–341.

6. Pastor, R.W., B.R. Brooks, and A. Szabo. 1988. An analysis of the accuracy of Langevin and molecular dynamics algorithms. Mol. Phys. 65: 1409–1419.

7. Loncharich, R.J., B.R. Brooks, and R.W. Pastor. 1992. Langevin dynamics of peptides: the frictional dependence of isomerization rates of N-acetylalanyl-N'-methylamide. Biopolymers. 32: 523–35.

8. Izaguirre, J.A., D.P. Catarello, J.M. Wozniak, and R.D. Skeel. 2001. Langevin stabilization of molecular dynamics. J. Chem. Phys. 114: 2090–2098.

9. Berendsen, H.J.C., J.P.M. Postma, W.F. van Gunsteren, A. DiNola, and J.R. Haak. 1984. Molecular dynamics with coupling to an external bath. J. Chem. Phys. 81: 3684.

10. Schneidman-Duhovny, D., M. Hammel, J.A. Tainer, and A. Sali. 2013. Accurate SAXS Profile Computation and its Assessment by Contrast Variation Experiments. Biophys. J. 105: 962–974.

11. Schneidman-Duhovny, D., M. Hammel, and A. Sali. 2010. FoXS: a web server for rapid computation and fitting of SAXS profiles. Nucleic Acids Res. 38: W540-544.

12. Schneidman-Duhovny, D., M. Hammel, J.A. Tainer, and A. Sali. 2016. FoXS, FoXSDock and MultiFoXS: Single-state and multi-state structural modeling of proteins and their complexes based on SAXS profiles. Nucleic Acids Res. 44: W424–W429.

13. Pelikan, M., G.L. Hura, and M. Hammel. 2009. Structure and flexibility within proteins as identified through small angle X-ray scattering. Gen Physiol Biophys. 28.
